# Transcriptomic stability or lability explains sensitivity to climate stressors in coralline algae

**DOI:** 10.1186/s12864-022-08931-9

**Published:** 2022-10-27

**Authors:** Tessa M. Page, Carmel McDougall, Ido Bar, Guillermo Diaz-Pulido

**Affiliations:** 1 Griffth University School of Environment and Science Nathan Campus, Griffith University, Nathan, QLD Australia; 2 Australian Rivers Institute Nathan Campus, Griffith University, Nathan, QLD Australia; 3 Coastal and Marine Research Centre Nathan Campus, Griffith University, Gold Coast, QLD Australia; 4 School of Ocean and Earth Science University of Southampton Waterfront Campus, National Oceanography Centre, Southampton, UK; 5 Centre for Planetary Health and Food Security Nathan Campus, Griffith University, Nathan, QLD Australia

**Keywords:** Coralline algae, Resistance, Global change, Transcriptomics, RNA-Seq

## Abstract

**Background:**

Crustose coralline algae (CCA) are calcifying red macroalgae that play important ecological roles including stabilisation of reef frameworks and provision of settlement cues for a range of marine invertebrates. Previous research into the responses of CCA to ocean warming (OW) and ocean acidification (OA) have found magnitude of effect to be species-specific. Response to OW and OA could be linked to divergent underlying molecular processes across species.

**Results:**

Here we show *Sporolithon durum*, a species that exhibits low sensitivity to climate stressors, had little change in metabolic performance and did not significantly alter the expression of any genes when exposed to temperature and pH perturbations. In contrast, *Porolithon onkodes*, a major coral reef builder, reduced photosynthetic rates and had a labile transcriptomic response with over 400 significantly differentially expressed genes, with differential regulation of genes relating to physiological processes such as carbon acquisition and metabolism. The differential gene expression detected in *P. onkodes* implicates possible key metabolic pathways, including the pentose phosphate pathway, in the stress response of this species.

**Conclusions:**

We suggest *S. durum* is more resistant to OW and OA than *P. onkodes*, which demonstrated a high sensitivity to climate stressors and may have limited ability for acclimatisation. Understanding changes in gene expression in relation to physiological processes of CCA could help us understand and predict how different species will respond to, and persist in, future ocean conditions predicted for 2100.

**Supplementary Information:**

The online version contains supplementary material available at 10.1186/s12864-022-08931-9.

## Background

Uncertainties associated with anthropogenic global change have presented challenges for predicting the persistence of species in the ocean. Transcriptomic profiling allows for investigation of molecular responses of organisms to stressors and can be informative in indicating mechanisms for resistance or adaptation [1] such as tolerance [2–4] and plasticity [3], or can indicate sensitivity [2–4]. Resistance and adaptation as responses to climate stressors can be measured at a molecular level with transcriptomics and can be seen through transcriptomic plasticity (i.e., shifting expression profile of transcriptome) or as a muted or dampened transcriptomic response [3, 5]. Phenotypic plasticity is a possible response to a changing environment, with the transcriptome being a phenotype that responds to environmental cues [3, 6], however, plasticity does not always indicate acclimatisation or adaptive strategy [7]. Environmental stressors can destabilise the transcriptome causing differential regulation of genes. This transcriptomic lability can be indicative of a deleterious stress outcome [3, 8–10]. Conversely, a muted or dampened transcriptional response, which we refer to as transcriptomic stability, can indicate resistance [3, 5, 11]. Transcriptomic stability associated with resistance to stressors has been documented in both gymnosperms (pines, [8, 9]) and angiosperms (tomato plants [10], and *Arabidopsis thaliana* [2]). However the prevalence of transcriptomic stability vs lability has not been investigated in one of the most important groups of coral reef organisms, crustose coralline algae (CCA). This may be particularly critical for understanding the molecular and cellular responses of marine algae to climate stressors and is of particular interest for those algae that play crucial ecological roles in coral reefs [12].

**Fig. 1:**
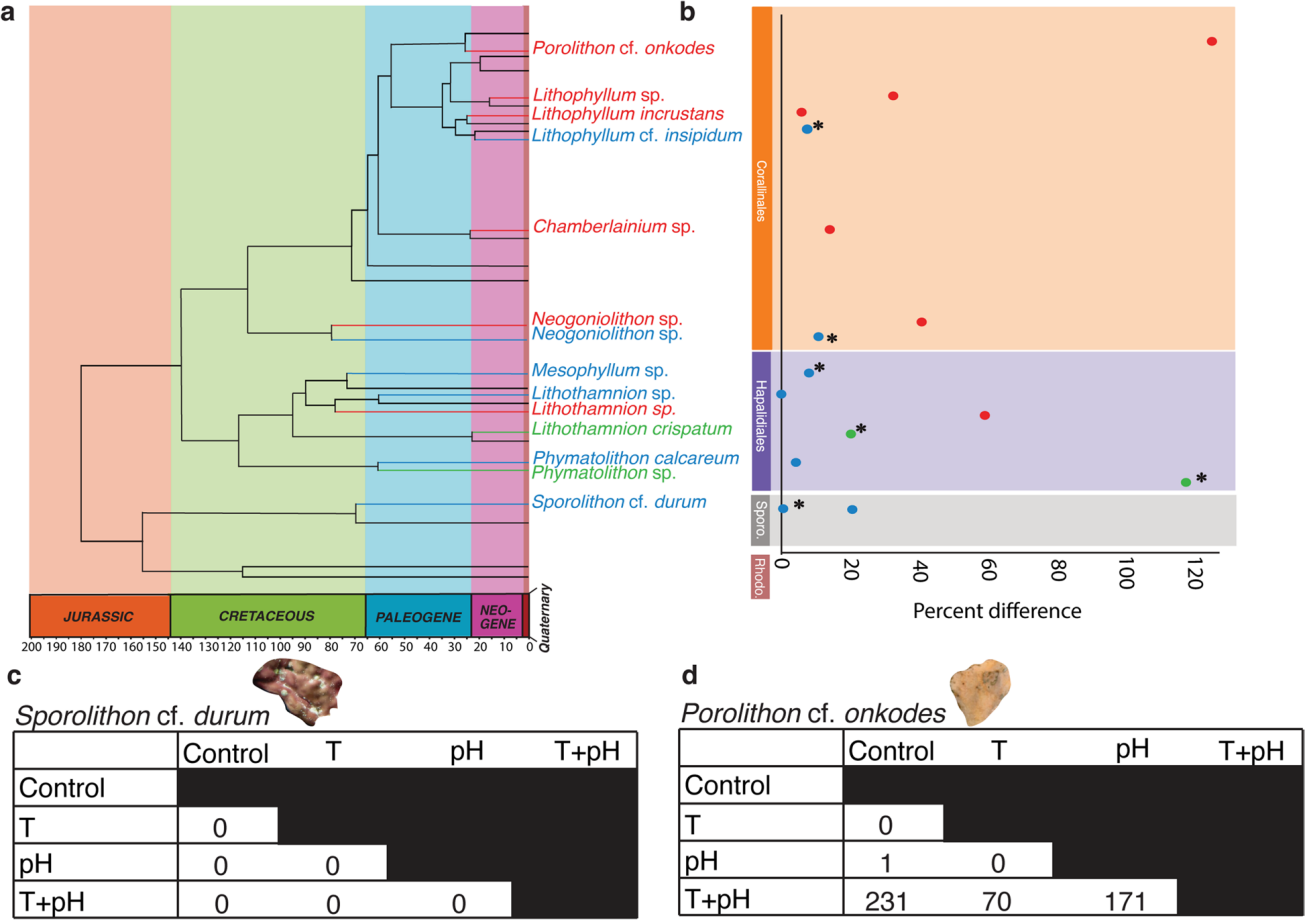
Variable responses of species from different orders of CCA to global change stressors from previous literature (**a,b**) and the current experiment (**c,d**). **a** Phylogenetic tree, adapted from V Peña, C Vieira, J Carlos Braga, J Aguirre, A Rösler, G Baele, O De Clerck and L Le Gall [17], showing different species across orders of CCA and their estimated divergence time (x axis). Species names are colour coded to show direction of response to elevated temperature + reduced pH; red – significant negative response; green – significant positive response; blue – no significant effect. Data was obtained from 9 studies (Additional file 1, Table S1). **b** Graphical representation of response data of species (Additional file 1, Table S1) from phylogenetic tree (**a**). Response is displayed as mean percent difference of photosynthetic rate or capacity per species. Points are colour coded to show direction of response to elevated temperature + reduced pH; red: significant negative response; green: significant positive response; blue: no significant effect. Asterisks signify results from studies using Pulse-Amplitude-Modulation fluorometry to indicate photosynthesis, whereas all other studies directly measured dissolved O_2_ in seawater. **c** Table of differentially expressed genes detected in pairwise comparisons across four treatments, control (27.2 ºC and 8.0 pH), T (29.5 ºC and 8.0 pH), pH (27.2 ºC and 7.7 pH), and T + pH (29.5 ºC and 7.7 pH), for *Sporolithon durum* and **d** *Porolithon onkodes*

CCA are important marine organisms because of their significance as ecosystem engineers (e.g., construction of coral reefs, coralligenous habitats, and maerl beds [13]), their positive role in ecological reef resilience by inducing settlement of coral larvae [14], and their contribution to the global carbon cycle [15]. Some CCA genera have persisted and diversified through times of elevated temperature and *p*CO_2_/reduced pH that equal or surpass levels projected for the year 2100 [16–18]. Previous experiments have found CCA to be negatively impacted by OW and OA [19–22], however, there is obvious variability in type and magnitude of response that seems to be species-specific. Whether this variability is found in molecular responses as well as previously measured physiological and biological responses remains unexplored. From previous literature, some species of CCA tend to be more resistant to OW and OA [23, 24], whereas other species seem to be more sensitive [19, 24–26] (Fig. 1a,b, Additional file 1, Table S1). Investigating the molecular basis of these responses could reveal the mechanisms by which resistance is obtained and facilitate comparisons between species responses to global change stressors.

To investigate this we exposed two species of CCA, *Sporolithon* cf. *durum* and *Porolithon* cf. *onkodes,* to differing levels of seawater temperature and pH, selected to reflect both current conditions and those projected for year 2100 [18]. Experiments were conducted for three months. Physiological (photosynthetic) responses were measured, and RNA sequencing analysis was used to investigate transcriptomic stability or lability as a means to propose resistance or sensitivity in CCA. Our findings reveal that OW and OA influence key physiological processes of CCA, at the level of both phenotype and gene expression, and magnitude of effect is species-specific, suggesting some species could be more resistant to global climate change than others.

## Results

*P. onkodes* showed transcriptomic lability, with 473 differentially expressed genes (DEGs) detected after three months in experimental treatments. No DEGs were detected in *S. durum* despite exposure to elevated temperature and *p*CO_2_, which we propose equates to transcriptomic stability. The transcriptional response in *P. onkodes* was only observed under the combined stressor treatment (T + pH; Fig. 1); only one gene (containing a lipoxygenase domain) was differentially expressed between the control and a single-stressor treatment (pH). The transcriptomic findings reflected physiological results, in which *S. durum* was proposed to be resistant to OW and OA in terms of survival and metabolic rates [23], whereas net photosynthesis of *P. onkodes* was significantly reduced under the combined treatment of T + pH (ANOVA, *F*_*1,16*_ = *4.782, p* = 0.046, Additional file 1, Figure S1, Table S2).

**Fig. 2:**
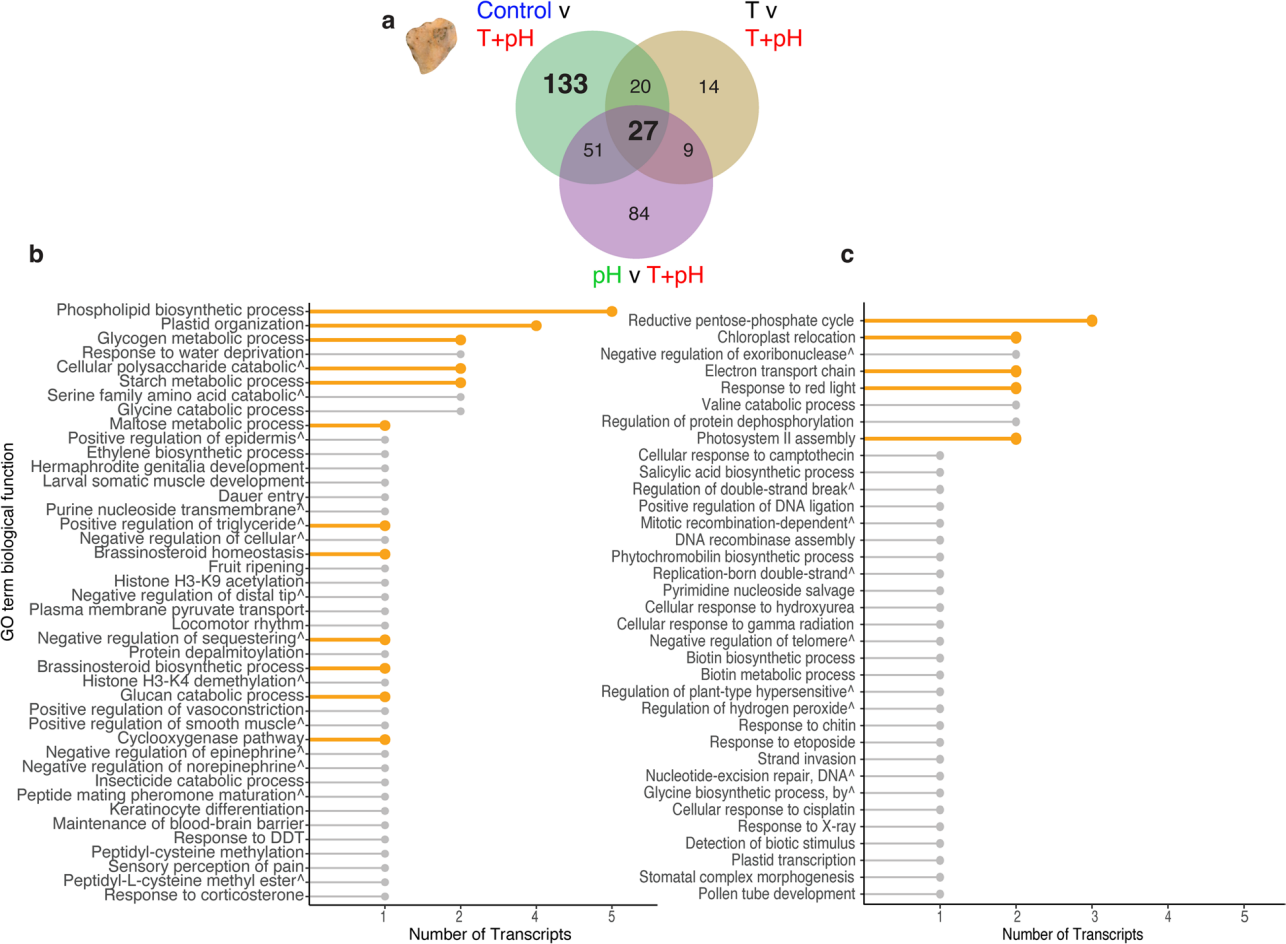
Unique and common differentially expressed genes found in pairwise comparisons between different experimental treatments. **a** Venn diagram of differentially expressed genes found in pairwise comparisons for *Porolithon onkodes* for all treatments compared to the T + pH treatment. 27 common transcripts were found to be differentially expressed (DE) across all comparisons. **b** Terminal nodes of overrepresented biological processes of the 133 transcripts that were found to be uniquely DE between the control treatment (27.2 ºC and 8.0 pH) and the T + pH treatment (+ 2.3 ºC and -0.3 pH units). Biological processes relevant to discussed (see text) physiological processes are highlighted in orange. The y-axis represents the gene ontology term for the biological process and the x-axis is the number of transcripts belonging to the process. **c** Terminal nodes of overrepresented biological processes of the 27 shared transcripts that were found to be commonly DE across all comparisons. Graph displays biological processes (y-axis) and number of transcripts per process (x-axis)

The transcriptional response of *P. onkodes* likely reveals the molecular mechanisms underlying the observed physiological response to stress. 133 DEGs were uniquely found in the T + pH vs control comparison, and 27 were commonly differentially expressed across all treatments when compared to T + pH (Fig. 2a). Functional overrepresentation analysis of the 133 DEGs revealed biological processes relating to catabolism and metabolism of polysaccharides, plastid organisation, and phospholipid biosynthesis, with the latter two containing largest number of transcripts, 4 and 5 respectively (Fig. 2b). 17 transcripts were related to processes involving carbohydrates and lipids. Transcripts found across all T + pH comparisons were primarily overrepresented in biological functions relating to carbon acquisition and metabolism (Fig. 2c).

**Fig. 3:**
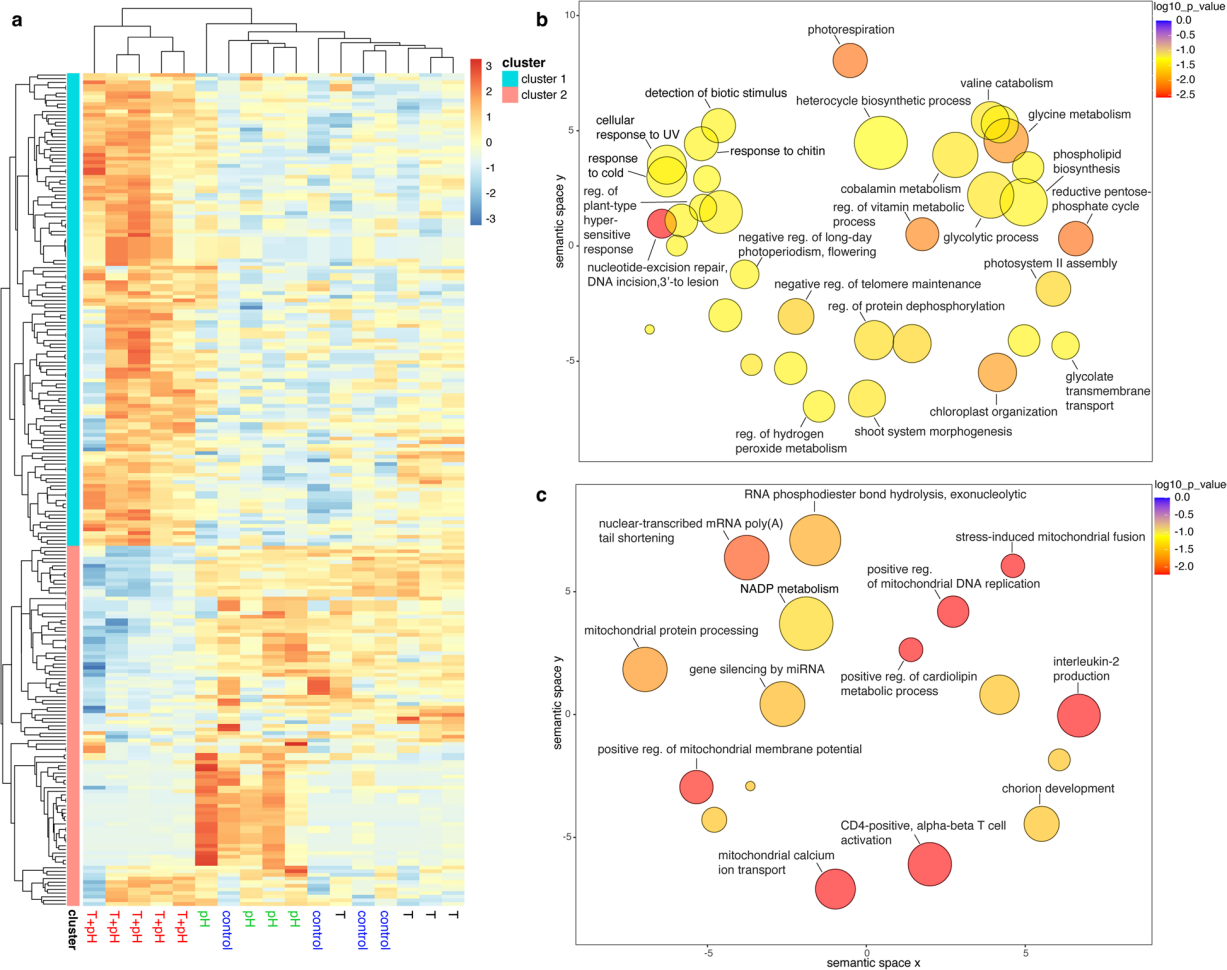
Patterns of differentially regulated gene expression across *Porolithon onkodes* experimental treatments. **a** The T + pH treatment results in significantly upregulated and downregulated transcripts when compared to other treatments (heatmap of log2-fold-change FDR values < 0.05). Experimental treatments are labelled at the bottom of the heatmap. Two main clusters corresponding primarily to upregulated (cluster 1) and downregulated (cluster 2) transcripts in the T + pH treatment are evident. **b** Overrepresented biological processes (terminal nodes; corrected *p*-values < 0.05) within cluster 1 transcripts include metabolism and catabolism, response to stimuli (biotic and abiotic), and regulation. Circle size indicates frequency of the gene ontology term in the UniProt database; colour indicates significance, on log_10_ *p* value scale. Axes have no intrinsic meaning; however, semantically similar gene ontology terms remain closely together in the plot. **c,** Biological processes corresponding to terminal nodes from overrepresentation analysis of transcripts found in cluster 2 include mitochondrial processes, chorion development, and immune response

The lack of significant differential gene expression or observed inactivity in *S. durum* was tested by creating a heatmap of genes that were expressed but not significantly (FDR > 0.05) (Supplemental Figure S3). Although *S. durum* showed no significant differentially regulated genes or DEGs, they were not found to be totally inactive (Supplemental Figure S3). Transcriptomic lability in *P. onkodes* was further observed in the patterns of differentially regulated genes within the T + pH treatment (Fig. 3a). Upregulated transcripts (130) were overrepresented in biological functional groups related to photorespiration, glycine metabolism, the reductive pentose phosphate cycle, chloroplast organisation, and nucleotide-excision repair (Fig. 3b). Downregulated transcripts (*n* = 99) were involved in biological functional groups related to the following mitochondrial processes: protein processing, positive regulation of membrane potential, stress-induced fusion, positive regulation of DNA replication, and calcium ion transport (Fig. 3c).

**Fig. 4:**
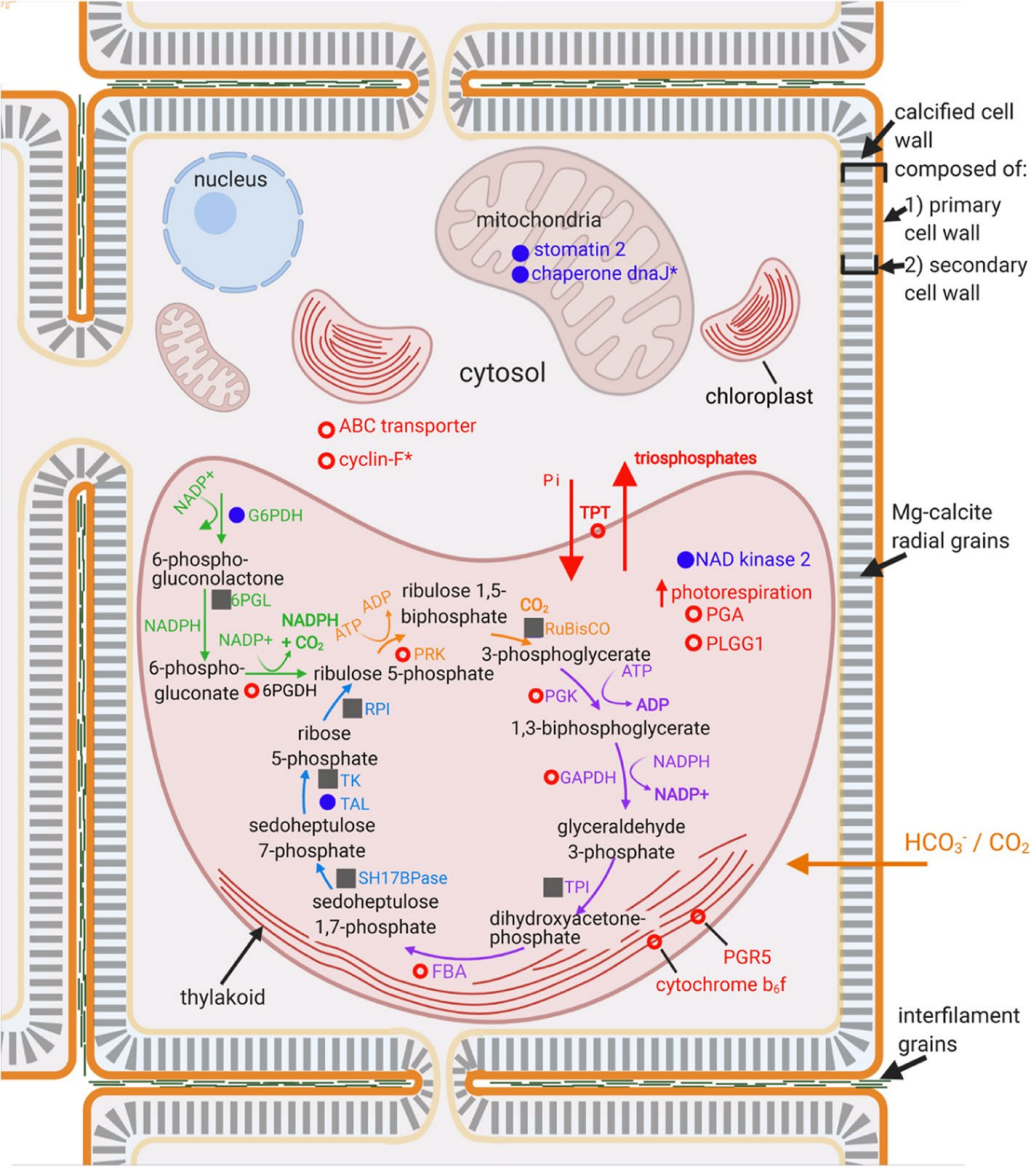
Conceptual model of the cellular pathways affected by global change stressors in *Porolithon onkodes*. Conceptual model shows proposed subcellular locations of differentially expressed genes and proposed pathway involvement. Expression levels of proteins and enzymes are based on results from differential expression and functional overrepresentation analyses of transcripts within clusters 1 and 2 from Fig. 3a. Red open circles denote significant (FDR < 0.05) upregulation and blue solid circles significant downregulation under the T + pH treatment. Grey squares denote transcripts that were found within the *P. onkodes* transcriptome and within edgeR differential expression analysis but were not found to be significantly differentially expressed. Asterisks (*) signify proteins that could have other subcellular localisations based on database (UniProt [27] and COMPARTMENTS) investigations. Enzymes encoded by transcripts from this study were found in the Calvin-cycle (orange), glycolysis (purple), and the pentose phosphate pathway, both the oxidative (green) and nonoxidative branch (blue), with these pathways being proposed to occur within the plastid/chloroplast of the algae. Definitions of abbreviations for proteins are found in Additional file 1, Table S4. HCO_3_^−^ and CO_2_ are proposed to enter into the cell and directly used as a substrate for photosynthesis and calcification. Conceptual model was created with BioRender.com

A large proportion (51 of 130, 39.2%) of the transcripts that were found to be upregulated in *P. onkodes* in the T + pH treatment encoded enzymes, and many of these are known components of the pentose phosphate pathway (PPP). This indicates that crucial metabolic processes of *P. onkodes* are affected by the synergistic effects of OW and OA. To visualise this, the proposed cellular locations of a subset of proteins encoded by transcripts from significantly enriched terminal biological processes from this study are shown in Fig. 4 (all proteins listed in Additional file 1, Table S3 and extended information in Additional File 2). Enzymes involved in the non-oxidative branch of the PPP were downregulated, whereas enzymes involved in the oxidative branch of the PPP were both downregulated (G6PDH and 6PGL) and upregulated (6PGDH). All differentially expressed enzymes involved in glycolytic reactions were upregulated (Fig. 4). Enzymes involved in Calvin-cycle specific reactions that were differentially expressed included PRK (significantly upregulated) and RuBisCO. Two proposed thylakoid membrane proteins (cytochrome b_6_f and PGR5) were significantly upregulated; both proteins play a role in photosynthesis with involvement in either or both photosystem complexes. The mitochondrial proteins stomatin-2 and chaperone protein dnaJ were significantly downregulated.

## Discussion

Researching molecular responses of CCA and how these responses relate to physiological measurements (e.g. photosynthesis and respiration) is central to understanding the impact of environmental stressors on CCA and more broadly coral reefs. The present study on two species of reef-building coralline algae, *S. durum* and *P. onkodes,* provides insight into biological processes that are likely to be altered in response to environmental stressors through measurements of gene expression and metabolic rates. In *P. onkodes*, transcripts found to be uniquely differentially expressed in T + pH were present in processes involving carbohydrates and lipids. Carbohydrates, specifically polysaccharides, have been suggested to play a role in the calcification process of CCA by acting as a matrix for biomineralization in their primary cell wall [28]. Changes in calcification rates observed in previous studies [19, 25, 29] could potentially be explained by alterations of expression of these transcripts, with negative implications for CCA biomineralization, reef cementation, and reef growth. Transcripts that were found commonly expressed across comparisons were related to functions such as carbon acquisition and metabolism, suggesting the combination of elevated temperature and reduced pH results in changes to crucial primary physical and chemical processes in *P. onkodes*. When investigating the differential expression of certain transcripts, it was found that the 99 downregulated transcripts were involved in biological functional groups relating to some mitochondrial processes. Mitochondria are the powerhouses of eukaryotic cells and a growing area of plant research involves linking mitochondrial function and composition to environmental stress response [30, 31]. Our finding of overrepresentation of mitochondrial-related processes in downregulated genes supports a role for mitochondria in the stress response and physiological processes of *P. onkodes*. Downregulation could be indicative of a negative effect on physiological processes, as was suggested in Antarctic algae in response to heat stress [32]. Two proposed proteins that play a role in photosynthesis were significantly upregulated in *P. onkodes*, while chaperone protein dnaJ was significantly downregulated. Generally, upregulation of genes involved in protective stress responses (dnaJ) can facilitate a faster and more efficient response [5]. Collectively, these results indicate that global climate change drivers have a significant impact on the energy cycle of *P. onkodes*. O_2_ production in *P. onkodes* decreased in response to T + pH, simultaneously we found enzymes involved in photorespiration were upregulated. We propose that reallocation of energy to photorespiration may have resulted in a decrease in the efficiency of photosynthesis, which was observed in the decrease in the rate of net photosynthesis/average O_2_ production (Additional file 1, Figure S1). Interestingly, in the present study, *S. durum* did not share a similar response to *P. onkodes* and instead had no DEGs after three months in treatment, possibly indicating some level of resistance in this species. The nature of the molecular response to OW and OA was unknown in CCA, but, as shown here, likely underlies potential resistance or susceptibility.

Our study demonstrates that transcriptional response differs between species, with CCA species showing differences in resistance and susceptibility to global change stressors, supporting our hypothesis that transcriptomic response and physiological responses are not uniform across CCA. To our knowledge, there are currently no published systematic studies that have specifically investigated differences in transcriptomic responses to stressors in multiple species of coralline algae, however physiological studies that are available suggest that response to stressors is species-specific (Fig. 1a,b). There are several factors that may explain the varying responses across species, including differences in anatomical and mineralogical features [33], variability in ecological niches (e.g., light and nutrient requirements, depth, hydrodynamics), and/or different evolutionary histories.

One possibility is the increased tolerance and a muted transcriptomic response in *S. durum* may be related to its more ancient evolutionary origin (the genus originated ~ 70 mya and has undergone little recent diversification [17]), where *Sporolithon* spp. persisted through periods of elevated ocean temperature and *p*CO_2_/reduced pH in the geological past (e.g. Paleocene-Eocene Thermal Maximum) that equalled or surpassed levels projected for the year 2100 [16–18]. In contrast, the labile transcriptomic response in *P. onkodes* may be more related to its recent evolutionary origin (the genus originated ~ 20 mya and has exhibited considerable recent diversification [17, 34]). A review discussing temperature tolerance in terrestrial plants indicated a more tolerant species, *Arabidopsis thaliana*, had a muted transcriptional response compared with a less tolerant species, *Sorghum bicolor* [2]. Interestingly, these two species have very different evolutionary histories, with the *Arabidopsis* genus having diverged ~ 43 mya [35] and the lineage containing *S. bicolor* estimated to have diverged between 3.9 – 2.4 mya [36]. This suggests that increased sensitivity in more recently derived groups may be a feature of Archaeplastida.

A stable or dampened transcriptional response in a tropical reef coral to high variable temperature environment has been found to be indicative of thermal tolerance [11], however, a muted response or lack of expression response could also be indicative of a stressor being mild [4]. In the current study, *S. durum* may have been unable to respond because the stressor (i.e. elevated temperature and/or reduced pH) was not strong, or perhaps not long, enough to induce a response in this species. This possibility still indicates a level of resistance in *S. durum*, however. Although we observed a muted or lack of apparent responsiveness in *S. durum*, there could be other significant posttranslational modifications occurring that we did not measure or analyse that could be indicative of stress in this species [37, 38], and future studies should consider measuring changes across multiple functional levels (e.g. transcriptome, proteome, metabolome). Furthermore, future studies should continue to investigate the responses of additional species of tropical CCA, across multiple clades and groups (e.g. Peña et al. 2021 [26] for temperate corallines) to investigate further species-specific response to climate stressors by systematically testing other possible contributing factors such as evolutionary history, acclimatisation history, and environmental history.

## Conclusions

This study is the first to reveal differentially expressed genes and pathways that underpin physiological responses of CCA to stressors, and to implicate genes involved in crucial chemical and physical processes (i.e., PPP, glycolysis, Calvin-cycle, and photorespiration). We propose that the differing transcriptional responses of CCA to global change drivers provides an explanation into the species-specific responses of CCA observed in previous studies. We suggest transcriptomic plasticity or lability, as seen in *P. onkodes*, is indicative of susceptibility to global change drivers, whereas transcriptomic stability, as seen in *S. durum*, is indicative of resistance in CCA taxa. Although it may be argued that plasticity is an expression of adaptation, that is not always the case [7] and at times plasticity can be maladaptive or not contribute to increased resistance in an organism [3, 7]. The findings from our study have implications for coral reef ecology worldwide. Our results indicate that *P. onkodes*, an abundant and significant reef-building species, may be negatively affected by predicted anthropogenic global change, with consequences for the distribution of the species and its contribution to reef cementation and resilience. In contrast, other tropical CCA species such as *S. durum*, although not currently major reef builders, may have the potential to thrive under predicted OW and OA scenarios.

## Methods

### Algae collection and experimental treatments

The two species used in this study were *S. durum* and *P. onkodes*. These two species were chosen for the following reasons: 1) They are abundant, reef building species, with *P. onkodes* being the primary reef building species in the Great Barrier Reef (GBR), Australia; 2) *S. durum* and *P. onkodes* have been found to have different sensitives to global change drivers from previous studies [19, 23]; and 3) they are two of the only four CCA species that currently have sequenced and assembled reference transcriptomes [39].

Adult fragments, ~ 3 cm^2^, of CCA from the species *S. durum* and *P. onkodes* (orange morph) were collected from lagoonal and reef crest sites surrounding Lizard Island, GBR, Australia. *S. durum* was collected between 7 – 9 m of depth and *P. onkodes* in depths no deeper than 3 m using hammer and chisel on SCUBA. After collection, algae fragments were transported to Lizard Island Research Station (LIRS) and held in an outdoor, flow through tank, maintaining similar seawater conditions to those measured at collection sites. All fragments were identified morphologically and anatomically, and representative samples have been stored in Dr. G.D-P.’s herbarium at Griffith University. Fragments were thoroughly cleaned of epiphytes within 24 h of collection. Fragments (*n* = 20 per species) were kept in control aquarium conditions for seven days at ambient temperature (26 ºC), pH (8.00), salinity (35 ppt), and natural light (30 – 50 µmol quanta m^−2^ s-^1^ for *S. durum* and 140 µmol quanta m^−2^ s-^1^ for *P. onkodes*) prior to being placed into experimental treatments. Light was measured using an underwater quantum sensor LI-192 connected to a light meter LI-250 (LI-COR, USA).

Following the seven days in common garden, fragments of CCA (20 per species) were divided across four treatments [“control” (unmanipulated seawater conditions, 27.2 ºC, 8.0 pH/450 µatm *p*CO_2_), “T” (elevated temperature and ambient pH, 29.5 ºC, 8.0 pH/450 µatm *p*CO_2_), “pH” (reduced pH and ambient temperature, 27.2 ºC, 7.7 pH/1000 µatm *p*CO_2_), or “T + pH” (elevated temperature and reduced pH, 29.5 ºC, 7.7 pH/1000 µatm *p*CO_2_)] with five biological replicates (*n* = 5) per treatment for a duration of three months. Elevated temperature and *p*CO_2_ levels were selected to closely mimic future increases in temperature and *p*CO_2_ expected by the end of this century under the representative concentration pathway 8.5 [18]. In treatments where temperature and/or *p*CO_2_ were manipulated, levels were increased over seven days reaching a 2.5 ºC increase in temperature and 0.3 unit decrease in pH (~ 1000 µatm *p*CO_2_). Temperature was maintained using titanium heaters (EcoPlus, Aqua Heat, 300 W), which were placed within each sump and were set to the desired temperature. Small 50 W glass heaters (Aqua One®) were placed in respective experimental tanks to correct for any heat loss when water moved from sump to experimental tanks. Temperature was adjusted daily across all treatments to mimic ambient temperature based on season (publicly available data from Australian Institute of Marine Science https://weather.aims.gov.au/#/station/1166) and + 2.5 ºC for elevated temperature treatments. pH/*p*CO_2_ was controlled by pH controllers (AquaController, Neptune Systems, USA) that injected either pure CO_2_ or ambient air into header sumps until set value was reached. Flow to each experimental tank was adjusted in the morning and evening, maintaining a flow rate of around 20 L h^−1^, which allowed for complete turnover in experimental tanks twice an hour. Submersible water pumps (Aqua One® 8 W) were placed in each experimental tank to ensure adequate water flow. Mortality and changes in CCA health were monitored and recorded throughout the experiment. Seawater temperature, pH (measured on total scale, pH_T_), and salinity were measured daily. Total alkalinity was measured every three days for the first week of the experiment and then weekly. Further detailed methods and seawater carbonate chemistry parameters are provided in an additional file (see Additional file 1, S1 Methods, Table S5).

### Physiological measurements

Photosynthesis and respiration were measured for *P. onkodes* (pink morph) following techniques described in TM Page and G Diaz-Pulido [23]. In summary, samples were incubated in sealed chambers for up to an hour under experimental conditions whilst continuously measuring % O_2_ using PreSens dipping O_2_ optodes (DP-PSt3) connected to a 10-channel trace O_2_ meter (OXY-10 SMA trace, G2, PreSens, Germany). Physiological data for *S. durum* was obtained from TM Page and G Diaz-Pulido [23] in which similar treatments of elevated temperature and *p*CO_2_ were used, however conducted over a longer period of time (five months versus three months in the current experiment).

### Molecular methodology

Fragments of CCA were thoroughly cleaned prior to sampling for molecular analysis under a microscope and all epiphytes, as far as possible, were removed. CCA were rinsed with filtered seawater and RNAlater® and then blotted with a kimwipe to remove bacterial film, following similar methods detailed in TM Page, C McDougall and G Diaz-Pulido [39]. RNA extraction procedure followed the method detailed in TM Page, C McDougall and G Diaz-Pulido [39]. RNA quantity was checked spectrophotometrically using an Invitrogen Qubit® Broad Range RNA kit, and ranged from 10.4 to 340 ng /µl. RNA was then used for preparation of cDNA libraries for sequencing and analyses. cDNA synthesis, library preparation, and sequencing followed the single-cell sequencing (CEL-Seq2) protocol detailed in Hashimonshony et al. 2016 [40] and McDougall et al. 2021 [41], which generates high sensitivity transcriptomes from low yield samples and utilised sample barcoding, 3’ end-tagging, and the inclusion of unique molecular identifiers. 25 ng of CCA RNA (*n* = 39) and 0.5 µl of ERCC spike-in (1:10,000 dilution) were added to an initial RNA/primer/ERCC/dNTP mix for each sample. Paired-end sequencing was performed at Ramaciotti Centre for Genomics, University of New South Wales, NSW, Australia. Customised sequencing was performed on a single lane of an Illumina NovaSeq 6000, sequencing 26 bp on read 1, and 100 bp on read 2. Further methods on processing of raw sequencing data and downstream analysis are outlined in Additional file 1, S2 Methods. Statistics for sequencing data are shown in Additional file 1, Table S6. RT-qPCR was conducted to validate gene expression results (Additional file 1, S2 Methods, Table S7).

### Systematic review of previous research and phylogenetic tree reconstruction

A systematic search for studies that investigated the metabolic responses of species of CCA to elevated temperature and ocean acidification (in combination) was conducted. The literature search was performed in the databases Google Scholar and Web of Science using keywords or topic codes such as ‘*crustose coralline algae*’ or ‘*coralline algae*’ in combination with ‘*photosynthesis, metabolic rates, ocean acidification, ocean warming, elevated temperature, reduced pH, elevated pCO*_*2,*_* global change or climate change*’. We focused on studies that measured photosynthesis using a similar methodology as that used in the current study and in TM Page and G Diaz-Pulido [23], however, as there were limited studies that fit our criteria, we supplemented the dataset with studies that used pulse amplitude modulated (PAM) fluorometry to determine photosynthetic capacity (Additional file 1, Table S1). The mean values of net photosynthesis or PAM fluorescence in the control and combined stressor treatments were obtained from publicly available datasets or, if results were only graphically represented, the built-in ruler and grids in Adobe Acrobat Pro DC *v* 2021.001.20138 (Adobe©) were used to obtain numerical values. Mean percent difference was calculated between control and the combined stressor treatment of elevated temperature and *p*CO_2_/reduced pH for each study (Additional file 1, Table S1). The absolute values of the percent differences were used in graphical representation. If studies manipulated other variables (i.e., nutrients or light), the control conditions for those variables were used. If studies measured over seasons, the average values from control and combined were taken across seasons. Mean percent differences were graphically represented adjacent to a reconstructed phylogenetic tree displaying species found in this review. The phylogenetic tree was adapted from V Peña, C Vieira, J Carlos Braga, J Aguirre, A Rösler, G Baele, O De Clerck and L Le Gall [17].

### Statistical analyses

Physiological data was analysed in R (v 3.6.1). Data were tested for normality through graphical analyses of residuals, using QQ normality plots, and using the Shapiro–Wilk test. Data were log transformed if they did not meet normality. Two-way ANOVAs were run for photosynthesis and respiration data using temperature and pH as fixed factors. If a significant interaction between treatments was identified, ANOVAs were followed by Tukey’s HSD post hoc pairwise comparisons. Differentially expressed genes (DEGs) were determined between treatments by a false discovery rate (FDR) cut-off of 5% and a log_2_-fold-change over 1.2.

## Supplementary Information

**Additional file 1.**

**Additional file 2: SI Dataset.** Table of all significantly (FDR < 0.05), differentially expressed genes (DEGs) from pairwise comparisons of experimental treatments from edgeR analysis for *Porolithon* cf. *onkodes*. Table includes *P.* cf. *onkodes* gene identifiers and values for log expression fold changes (logFC), log counts per million (logCPM), *F *statistic, *p* value, false discovery rate (FDR; *p* adjusted by the Benjamini-Hochberg procedure) for pairwise comparisons of each treatment combination. Treatment comparisons are listed as follows: control (27.2 ºC + 8.0 pH), T (29.5 ºC + 8.0 pH), pH (27.2 ºC + 7.7 pH), and T+pH (29.5 ºC + 7.7 pH). Annotations for DEGs that returned BLASTX similarity search hits are given. If transcript didn't return a hit, no annotation is given and cell is left blank.

## Data Availability

The data discussed in this publication have been deposited in NCBI’s Gene Expression Omnibus [42] and are accessible through GEO Series accession number GSE211882 (https://www.ncbi.nlm.nih.gov/geo/query/acc.cgi?acc=GSE211882). The datasets supporting the conclusions of this article are available in the Open Science Framework repository under the project “Transcriptomic responses of coralline algae to global change stressors”, 10.17605/OSF.IO/2NKR4 https://osf.io/2nkr4/. And, within the supplemental material (Additional files 1 & 2) provided with this article. Reference transcriptomes were created using transcriptomes for *P. onkodes* and *S. durum* (NCBI BioProject PRJNA518156, accession numbers GHIN00000000.1 and GHIO00000000.1 for *Sporolithon* cf. *durum* and *Porolithon* cf. *onkodes*, respectively).

## Abbreviations

CCA: Crustose coralline algae
OW: Ocean warming
OA: Ocean acidification
DEGs: Differentially expressed genes
*p*CO_2_: Partial pressure of carbon dioxide
ANOVA: Analysis of variance
FDR: False discovery rate
PPP: Pentose phosphate pathway
G6PDH: Glucose-6-phosphate 1-dehydrogenase
6PGL: 6-Phosphogluconolactonase
6PGDH: 6-Phosphogluconate dehydrogenase
RuBisCO: Ribulose-1,5-biphosphate carboxylase/oxygenase
PRK: Phosphoribulokinase
PGR5: Proton gradient regulation 5
GBR: Great Barrier Reef
LIRS: Lizard Island Research Station
PAM: Pulse amplitude modulated
