# Supplementary material for: Transcriptomic stability or lability explains sensitivity to climate stressors in coralline algae
Source: BMC Genomics. 2022 Oct 27;23:729. doi: 10.1186/s12864-022-08931-9 (PMC9615231; doi:10.1186/s12864-022-08931-9)
Supplement: Supplementary file 1 — Additional file 1. [file 12864_2022_8931_MOESM1_ESM.docx]

Additional File 1

Supplemental Information for:

Transcriptomic stability or lability explains sensitivity to climate stressors in coralline algae

Tessa M. Page^*^, Carmel McDougall, Ido Bar, Guillermo Diaz-Pulido^*^

Corresponding authors:

Tessa M Page: tessa.page@griffith.edu.au or Guillermo Diaz-Pulido: g.diaz-pulido@griffith.edu.au

Additional File 1, Supplementary Methods

**S1 Methods**

**Seawater chemistry**

Temperature and seawater pH (measured on total scale, pH_T_) were measured twice per day, at 08:00 and 15:00 in each experimental tank using a pH electrode with integrated temperature probe (Mettler Toledo, InLab Routine Pro) attached to a pH meter (Mettler Toledo, SevenGo Duo SG98). The pH electrode was calibrated on the total scale using Tris-HCl buffers [1]. Salinity was measured once a day using a conductivity meter (Mettler Toledo, SevenGo Pro). Total alkalinity (*A*_T_) was measured every 3 days for the first week of the experiment, and then every 6 – 7 days following using potentiometric titration on an automatic titrator (Mettler Toledo, T50) following standard operating procedures 3b [1]. pH_T_, *A*_T_, temperature, and salinity were used to calculate the remaining carbonate chemistry variables using the seacarb package version 3.2.12 [2] in the statistical software R version 3.5.1 (Table S5). High-Mg calcite was calculated for a 16.4% MgCO_3_, following method described in G Diaz-Pulido, KRN Anthony, DI Kline, S Dove and O Hoegh-Guldberg [3].

**S2 Methods**

**Sequencing preparation, sequencing, and differential expression analyses**

The top, pigmented layer of the CCA fragments was collected under a microscope using sterile razors and placed directly into RNAlater®. Care was taken to avoid collection of any endolithic organisms when obtaining samples. Samples were left at 4 ºC for 24 hrs and then transferred to -20 ºC until processed. RNA extraction procedure followed the method detailed in TM Page, C McDougall and G Diaz-Pulido [4]. RNA quantity was checked spectrophotometrically using an Invitrogen Qubit® Broad Range RNA kit, and ranged from 10.4 to 340 ng /µl. One RNA sample of *S. durum* yielded an undetectable amount of RNA and therefore was not used for further analyses. A random selection of samples, 2 – 3 samples from each treatment and species, were tested using the 4200 TapeStation System to ensure RNA quality and absence of degradation and contamination. RNA was then used for preparation of cDNA libraries for sequencing and analyses.

cDNA synthesis, library preparation, and sequencing followed the single-cell sequencing (CEL-Seq2) protocol detailed in Hashimonshony et al. 2016 [5] and McDougall et al. 2021 [6], which generates high sensitivity transcriptomes from low yield samples and utilised sample barcoding, 3’ end-tagging, and the inclusion of unique molecular identifiers. 25 ng of CCA RNA (*n* = 39) and 0.5 µl of ERCC spike-in (1:10,000 dilution) were added to an initial RNA/primer/ERCC/dNTP mix for each sample. Paired-end sequencing was performed at Ramaciotti Centre for Genomics, University of New South Wales, NSW, Australia. Customised sequencing was performed on a single lane of an Illumina NovaSeq 6000, sequencing 26 bp on read 1, and 100 bp on read 2. A technical issue was identified for one sample for *P. onkodes,* and this sample was not considered in downstream analyses after sequencing.

Raw sequencing data were processed using the Illumina BCL2FASTQ software, using default settings but with a minimum trimmed read length of 15. Quality control was performed on the FastQ files using FastQC (v 0.11.3, Babraham Bioinformatics). FastQ files have been deposited on an OSFHome repository (<https://osf.io/2nkr4/>). Reference transcriptomes were created using transcriptomes for *P. onkodes* and *S. durum* (NCBI BioProject PRJNA518156, accession numbers GHIN00000000.1 and GHIO00000000.1 for *S.* cf. *durum* and *P.* cf. *onkodes*, respectively) [4]. PolyA sequences were removed from reference transcriptomes using prinseq [7]. ERCC sequences, without polyA’s, were appended to the transcriptome files. Bowtie indices were generated using bowtie [8] v 2 – 2.0.2. The resulting FASTA files were used to generate files mimicking gene transfer format (gtf) files following protocol laid out in McDougall et al. 2021 [6]. These “fake” gtf files were then used in the publicly available CEL-Seq-pipeline (<https://github.com/yanailab/CEL-Seq-pipeline>). Prior to mapping, sequence quality across read was evaluated. Reads were then trimmed at 60 bp (using the 'cut_length' parameter in the CEL-Seq pipeline) due to quality dropping beyond 66bp, which maximised mapping specificity while minimising the chance for mismatches due to sequence error. Demultiplexed and trimmed reads were mapped against the reference transcriptomes and read counts per transcript were generated. On average, 67 and 53% of the sequence reads mapped successfully to the reference transcriptomes of *S. durum* and *P. onkodes*, respectively. Samples that had less than 900,000 demultiplexed reads and less than ~ 40% mapped reads were removed from further analyses (*n* = 2 for *P. onkodes*) (Table S6). Counts were imported into R (v 3.6.1) and corrected to account for the possibility of transcripts getting the same UMI. To correct for this and to convert UMI counts to transcript numbers the binomial method outlined in D Grün, L Kester and A van Oudenaarden [9] was used. Transcripts were filtered using a cut-off of 5 counts per transcript across all samples to remove transcripts with very low counts. After filtering, 24% of transcripts were retained for *S. durum* and 20% for *P. onkodes*.

Differential gene expression analysis for each species was performed using the Bioconductor software package edgeR, v 3.16.8 [10]. Negative binomial (NB) generalised linear models (GLMs) were fitted to transcript counts and common dispersions (0.169 and 0.293 for *P. onkodes* and *S. durum*, respectively) were estimated [10]. A design matrix of the experiment was used for analysis to identify expression in response to treatment. Quasi-likelihood (QL) F-tests were used in determining differential expression using default settings and the parameter ‘robust=TRUE’ to identify genes that were outliers from the mean-NB dispersion trend. Pairwise comparisons were conducted on specified constructed parameters (i.e., treatments) where genes that exhibited positive or negative log-fold changes were identified. Differentially expressed genes (DEGs) between treatments with a false discovery rate (FDR) cut-off of 5% and a log_2_-fold-change above 0.3 (log_2_FC>0.3, i.e., a fold-change over 1.2), were extracted and the datasets concatenated to use for downstream analyses and visualisations. FDR correction was applied using the Benjamini-Hochberg method on the *p*-values [10].

Visualisation of DEGs was performed using variance stabilising transformed (vst) counts from edgeR. Principal component analyses were performed for each species to explore the variation in DEGs within species (Figure S2). Heatmaps were constructed for DEGs from each species using the R package pheatmap (v 1.0.12) [11]. An FDR cut-off of 0.05 was used when creating the heatmap. For *S. durum*, because they displayed 0 DEGs (FDR < 0.05), a heatmap was created with expressed genes that were not significant (FDR > 0.05) to visualise if they were totally inactive or not (see Figure S3). Functional overrepresentation analysis of differentially expressed transcripts was performed in the Cytoscape [12] plugin BiNGO [13], where hypergeometric tests of gene ontology (GO) categories, specifically “biological process”, were used, with the annotated transcriptome of *P. onkodes* as a ‘background’, and a *p*-value (Benajmini-Hochberg FDR correction) cut-off of 0.01. BiNGO also allowed for identification of terminal node biological processes. REVIGO [14] was used to summarise and visualise gene ontology terms obtained from enrichment analysis.

Similarity searches for DEGs were conducted using NCBI’s (National Center for Biotechnology Information) Basic Local Alignment Search Tool (BLAST) [15] using the default e-value cut-off of 0.01. In order to identify KEGG pathway components we used the KEGG Mapper – Reconstruct Pathway tool [16]. KEGG annotations were obtained for all expressed and differentially expressed genes for each species using previous KEGG annotations from previously annotated transcriptomes [4]. Proposed cellular locations and pathway involvement of DEGs used in Figure 4 were based on BLASTX similarity searches and KEGG Mapper Reconstruction results. Subcellular localisations were obtained through BLASTX and further checked on the subcellular localisation database, COMPARTMENTS (https://compartments.jensenlab.org/).

## RT-qPCR validation of expression results from CEL-Seq analysis

For qPCR validation of CEL-Seq edgeR expression analysis, reference genes and genes of interest (GOI) were chosen from edgeR normalised reads data. To obtain candidate reference genes we performed reciprocal BLAST analyses with the transcriptomes of *S. durum* and *P. onkodes* to find orthologues of reference genes that have been used with other species of red algae (i.e., the fleshy, red alga *Pyropia haitanensisi*), such as ß-tubulin and ubiquitin conjugating enzyme (UBC), and glyceraldehyde 3-phosphate dehydrogenase (GAPDH) [17]. We also assessed whether better-performing reference genes could be identified from our transcriptomics data. To do this we used a coefficient variation (CV) model to assess the degree of variation for each gene from edgeR normalised reads data, and selected several genes with low variation to identify candidate reference genes [18]. To calculate the CV, we found the ratio of the standard deviation to the mean of each gene, high CVs indicate more variation in expression of a gene, whereas low CVs indicate low variation. Reference genes were chosen if CV < 0.47 and if they had a low standard deviation. GAPDH was only used as a reference gene for *S. durum* as it was found to have a low CV, however, GAPDH was used as a GOI for *P. onkodes* because it was found to be significantly differently expressed, with a high CV.

Primers for GOIs were obtained from exploring *P. onkodes* edgeR data for differentially expressed genes of potential biological interest (e.g., heat-shock proteins, photosystem genes), and then performing reciprocal blast in the *S. durum* transcriptome to find orthologues. Once genes were selected, primer sets were designed for reference genes and GOIs using Primer3 [19].

To test and optimise primer sets, cDNA was synthesised using Superscript III reverse transcriptase (Invitrogen™) from DNase treated RNA of *S. durum* and *P. onkodes* and pooled for each species. Pooled cDNA was used as a template for PCR. PCRs using a thermal gradient (55 ºC – 65 ºC) were conducted to test primers and identify optimal annealing temperatures of primer sets. PCRs were run with 0.5 µL of forward and reverse primer (2.5 µM), 4.5 µL QuantiNova SYBR^®^ Green PCR Master Mix, 3.5 µL DNase/RNAse-Free H_2_O (PCR grade), and 1 µL cDNA pooled template (1:30 dilution). The thermal profile for PCR was 95 ºC for 2 min, followed by 60 cycles of 95 ºC for 5 s, thermal gradient (55 ºC – 65 ºC) for 30 s, and 60 ºC for 10 s. PCRs were assessed by gel electrophoresis on a 2.5% agarose gels.

Subsequent RT-qPCR (CFX96 Touch^TM^, Real-Time PCR Detection System, Bio-Rad) validation of reference genes and GOI was performed (Table S7). Optimal dilution for each gene, primer efficiencies, and coefficient of determination (R^2^) were obtained from serial dilutions of standard curves for each primer set (Table S7). The thermal profile for RT-qPCR was 95 ºC for 2 min, followed by 60 cycles of 95 ºC for 5 s, annealing temperature for specific primer set (identified from PCR) for 30 s, and 60 ºC for 10 s, followed by a melt curve (65 ºC to 95 ºC in 0.5 ºC increments for 5 s at each increment). RT-qPCR was then run to validate differential expression results of RNA-Seq data. Expression analysis through RT-qPCR was conducted on all individual, experimental samples for each species. No template controls were included for each primer set and each sample and reactions were carried out in technical and biological triplicates. The most suitable reference genes for expression analysis were determined through analysis of geometric means in geNorm [20]. For *P.* *onkodes* heme oxygenase and ß-tubulin were determined to be the most suitable reference genes and for *S. durum* GAPDH was identified as the most suitable reference gene (Table S7). Expression analysis was carried out in BioRad’s CFX Maestro Software, log_2_ ∆∆Ct values (relative expression) for each sample were calculated and *p* values obtained to assess significant differential expression across treatments and individuals (Figures S4 and S5), values were normalised to reference genes and predetermined efficiencies of each primer were entered based on standard curves (Table S7).

**Supplementary Figures and Tables**

Figure S1. Effect of global change drivers on metabolic processes of *Sporolithon* cf. *durum* and *Porolithon* cf. *onkodes*. **a**, Graph obtained from TM Page and G Diaz-Pulido [21] of the effect of experimental treatment on the mean amount of O_2_ produced or consumed by *S.* cf. *durum* after 5 months in treatment. Each bar represents mean O_2_ produced or consumed ± standard error (SE), n = 5 -6. **b**, The effect of experimental treatment on the mean amount of O­_2_ produced and consumed by *P.* cf. *onkodes* after 3 months in treatment, values are means ± SE, n = 5. Physiological measurements of *P. onkodes* were normalised to surface area obtained through aluminium foil technique [22]. Significant differences (*p* < 0.05), resulting from Tukey HSD postdoc pairwise comparisons, are indicated by different lowercase letters.

Figure S2. Principal component analysis run on a, *Sporolithon* cf. *durum* and b, *Porolithon* cf. *onkodes*. Blue squares indicate the “control” treatment (8.0 pH and 27.2 ºC), black circles the “T” temperature (8.0 pH and 29.5 ºC), green triangles “pH” treatment (7.7 pH and 27.2 ºC), and the red plus sign the “T+pH” treatment (7.7 pH and 29.5 ºC).

Figure S3. A heatmap of differentially expressed genes from *Sporolithon* cf. *durum* based on *p* values < 0.05, without FDR adjustment. Heatmap shows expression of genes in *S.* cf. *durum*, however, these were not significant. Treatment labels correspond to the following treatments: control (27.2 ºC and 8.0 pH), T (29.5 ºC and 8.0 pH), pH (27.2 ºC and 7.7 pH), and T+pH (29.5 ºC and 7.7 pH).

Figure S4. RT-qPCR validation graphs for *Porolithon* cf. *onkodes*. * denotes significant relative expression compared to a control sample (in bold). Values are the log_2_ relative expression (∆∆Ct) of genes using two reference genes (DN95780 and DN76782) ± normalised standard error of the mean (SEM). a) Transcript likely encoding for Hsp33 (BLASTX); b) transcript likely encoding photosystem II CP47 (BLASTX); and c) transcript likely encoding GAPDH (BLASTX).

Figure S5. RT-qPCR validation graphs for *Sporolithon cf. durum*. * denotes significant expression compared to a control sample (in bold). Values are the log^2^ relative expression (∆∆Ct) of genes using one reference (DN199936) ± normalised standard error of the mean (SEM). a) Transcript likely encoding for serine/threonine protein phosphate (BLASTX); b) transcript likely encoding transcript likely encoding Hsp33 (BLASTX); and c) transcript likely encoding acetyl-CoA (BLASTX).

Table S1. Metabolic responses of crustose coralline algae to the combined effects of elevated temperature and *p*CO_2_/reduced pH, as reported in previous studies. Species that were used in the phylogenetic tree are bolded, all studies in this table were used in the response graph in Figure 1. Green = positive response to elevated temperature and *p*CO_2_/reduced pH, red = negative response to elevated temperature and *p*CO_2_/reduced pH, and blue = no response. Included within the table are species names, order, estimated first occurrence of genera (obtained from V Peña, C Vieira, J Carlos Braga, J Aguirre, A Rösler, G Baele, O De Clerck and L Le Gall [23]), levels of temperature and *p*CO_2_/pH relative to current at time of divergence, species collection location, methodology of metabolic rate measurement, calculated mean value for control and elevated temperature and *p*CO_2_/reduced pH treatments, and respective study. PAM = Pulse Amplitude Modulation Fluorometry. GBR = Great Barrier Reef, Australia

| Species | Order | Divergence time *ca.* (mya) | | Historic level of TºC + *p*CO_2_/pH | | Location | | Methodology | | Mean at control | | Mean at high | | Study | |  |  |
| --- | --- | --- | --- | --- | --- | --- | --- | --- | --- | --- | --- | --- | --- | --- | --- | --- | --- |
| *Porolithon* cf. *onkodes* | *corallinales* | | 22 | | lower | | GBR | | Dissolved O_2_ | | 21 | | -5 | | KRN Anthony, DI Kline, G Diaz-Pulido, S Dove and O Hoegh-Guldberg [24] | | |
| *Porolithon* cf. *onokdes* | *corallinales* | | 22 | | lower | | GBR | | PAM | | 513 | | 506 | | E Bergstrom, A Ordoñez, M Ho, C Hurd, B Fry and G Diaz-Pulido [25] | | |
| *Chamberlainium*  sp. | *corallinales* | | 39 | | lower | | Korea | | Dissolved O_2_ | | 0.35 | | 0.4 | | J-H Kim, N Kim, H Moon, S Lee, SY Jeong, G Diaz-Pulido, MS Edwards, J-H Kang, EJ Kang, H-J Oh, et al. [26] | | |
| *Lithophyllum cabiochae* | *corallinales* | | 27 | | lower | | France | | Dissolved O_2_ | | 0.9 | | 0.61 | | S Martin, S Cohu, C Vignot, G Zimmerman and J-P Gattuso [27] | | |
| *Lithophyllum incrustans* | *corallinales* | | 27 | | lower | | France | | Dissolved O_2_ | | 1.78 | | 1.68 | | ZN Qui-Minet, J Coudret, D Davoult, J Grall, M Mendez‐Sandin, T Cariou and S Martin [28] | | |
| *Lithophyllum* cf. *insipidum* | *corallinales* | | 27 | | lower | | GBR | | PAM | | 441 | | 464 | | E Bergstrom, A Ordoñez, M Ho, C Hurd, B Fry and G Diaz-Pulido [25] | | |
| *Neogoniolithon* sp. | *corallinales* | | 105 | | higher | | Mexico | | Dissolved O_2_ | | 2.91 | | 1.73 | | RM Vásquez-Elizondo and S Enríquez [29] | | |
| *Neogoniolithon fosliei* | *corallinales* | | 105 | | higher | | GBR | | PAM | | 406 | | 447 | | E Bergstrom, A Ordoñez, M Ho, C Hurd, B Fry and G Diaz-Pulido [25] | | |
| *Phymatolithon lusitanicum* | *hapalidales* | | 60 | | lower | | Portugal | | Dissolved O_2_ | | 1.1 | | 2.4 | | L Sordo, R Santos, I Barrote and J Silva [30] | | |
| *Phymatolithon calcareum* | *hapalidales* | | 60 | | lower | | France | | Dissolved O_2_ | | 1.7 | | 1.8 | | ZN Qui-Minet, J Coudret, D Davoult, J Grall, M Mendez‐Sandin, T Cariou and S Martin [28] | |  |
| *Mesophyllum / Melyvonnnea sp.* | *hapalidales* | | 71 | | higher | | GBR | | PAM | | 472 | | 495 | | E Bergstrom, A Ordoñez, M Ho, C Hurd, B Fry and G Diaz-Pulido [25] | |  |
| *Lithothamnion* sp. | *hapalidales* | | 75 | | higher | | Mexico | | Dissolved O_2_ | | 0.48 | | 0.19 | | RM Vásquez-Elizondo and S Enríquez [29] | |  |
| *Lithothamnion corallioides* | *hapalidales* | | 75 | | higher | | France | | Dissolved O_2_ | | 2.15 | | 2.15 | | ZN Qui-Minet, J Coudret, D Davoult, J Grall, M Mendez‐Sandin, T Cariou and S Martin [28] | |  |
| *Lithothamnion proliferum* | *hapalidales* | | 75 | | higher | | GBR | | PAM | | 511 | | 412 | | E Bergstrom, A Ordoñez, M Ho, C Hurd, B Fry and G Diaz-Pulido [25] | |  |
| *Lithothamnion crispatum* | *hapalidales* | | 75 | | higher | | Brazil | | PAM | | 0.456 | | 0.55 | | PT Muñoz, CA Sáez, MB Martínez-Callejas, MR Flores-Molina, E Bastos, A Fonseca, CFD Gurgel, JB Barufi, L Rörig, JM Hall-Spencer, et al. [31] | |  |
| *Sporolithon* cf. *durum* | *sporolithales* | | 137 | | higher | | GBR | | Dissolved O_2_ | | 2.3 | | 1.8 | | TM Page and G Diaz-Pulido [21] | |  |
| *Sporolithon* cf. *durum* | *sporolithales* | | 137 | | higher | | GBR | | PAM | | 514 | | 513 | | E Bergstrom, A Ordoñez, M Ho, C Hurd, B Fry and G Diaz-Pulido [25] | |  |

Table S2. Results of two-way analysis of variance (ANOVA) for the effects of temperature and pH (*p*CO_2_) on the metabolic rates of *Porolithon* cf. *onkodes* (from the current experiment) and *Sporolithon* cf. *durum* (data acquired from Page & Diaz-Pulido, 2020).

|  | Oxygen produced (µmol O_2_ cm^-2^ h^-1^) | | | | | Oxygen consumed (µmol O_2_ cm^-2^ h^-1^) | | |
| --- | --- | --- | --- | --- | --- | --- | --- | --- |
| *Porolithon* cf. *onkodes* | Two-way ANOVA | Df | MS | *F* | *p* | MS | *F* | *p* |
|  | Temperature | 1 | 0.073 | 4.443 | 0.054 | 0.015 | 0.294 | 0.595 |
|  | pH | 1 | 0.000 | 0.000 | 0.988 | 0.021 | 0.405 | 0.534 |
|  | Temperature * pH | 1 | 0.078 | 4.782 | 0.046 | 0.204 | 3.938 | 0.065 |
|  | Residuals | 16 | 0.016 |  |  | 0.052 |  |  |
| *Sporolithon* cf. *durum* | Temperature | 1 | 0.056 | 0.439 | 0.516 | 3.026 | 1.830 | 0.192 |
|  | pH | 1 | 1.217 | 9.560 | 0.006 | 0.495 | 0.299 | 0.591 |
|  | Temperature * pH | 1 | 0.085 | 0.671 | 0.423 | 0.113 | 0.068 | 0.796 |
|  | Residuals | 18 | 0.127 |  |  | 1.654 |  |  |

*Oxygen produced or consumed was normalised to surface area (cm^2^) for *Porolithon* cf. *onkodes* and to the ash-free dry weight (g) of individual fragments for *S.* cf. *durum*.

Table S3. Table of all proteins found to belong to terminal node biological processes from functional enrichment analysis. Table contains transcript/protein ID, top BLASTX similarity search result, proposed cell location based on BLASTX similarity search, expression pattern of transcript/protein based on ANOVA-like test in edgeR comparing all treatment combinations, cluster of heatmap (Figure 3a), description of terminal node biological process obtained through enrichment analysis, and whether or not the transcript/protein was found to be unique to a specific biological process. All BLASTX hits were with red algae unless otherwise specified within table.

| Transcript /protein ID | BLASTX similarity result | Proposed cell location | Expression pattern | Heatmap cluster | Description of biological process | Unique to process |
| --- | --- | --- | --- | --- | --- | --- |
| DN72649_c1_g2_i2 | phospho-ribulokinase | chloroplast | upregulated | 1 | detection of biotic stimulus, heterocycle biosynthetic process, reductive pentose-phosphate cycle, photosystem II assembly, chloroplast organisation, shoot system morphogenesis, regulation of hydrogen peroxide metabolism, regulation of protein dephosphorylation, regulation of plant-type hypersensitive response, response to chitin | N |
| DN74711_c1_g3_i1 | green algae hit, Trebouxia 2Fe-2S ferredoxin-like |  | upregulated | 1 | heterocycle biosynthetic process, reductive pentose-phosphate cycle | N |
| DN70344_c0_g1_i2 | 6-phosphogluconate dehydro-genase NAD-binding domain-containing protein | cytosol | upregulated | 1 | valine catabolic process | Y |
| DN74502_c6_g1_i1 | serine-pyruvate amino-transferase |  | upregulated | 1 | glycine metabolic process | Y |
| DN74877_c1_g1_i1 | glyceralde-hyde-3-phosphate dehydro-genase | chloroplast | upregulated | 1 | heterocycle biosynthetic process, reductive pentose-phosphate cycle, negative regulation of telomere maintenance, nucleotide-excision repair, DNA incision,3’-to lesion | N |
| DN76886_c0_g1_i1 | soybean hit, putative oxido-reductase |  | upregulated | 1 | valine catabolic process | Y |
| DN70418_c0_g2_i2 | PsbB mRNA maturation factor Mbb1 | chloroplast | upregulated | 1 | heterocycle biosynthetic process, photosystem II assembly, chloroplast assembly, shoot system morphogenesis, regulation of protein dephosphorylation | N |
| DN75582_c0_g4_i1 | fructose-bisphosphate aldolase |  | upregulated | 1 | glycolytic process | Y |
| DN75582_c0_g4_i2 | fructose-bisphosphate aldolase |  | upregulated | 1 | glycolytic process | Y |
| DN74152_c0_g1_i4 | zinc finger protein 771, C2H2-type |  | upregulated | 1 | heterocycle biosynthetic process | Y |
| DN71318_c0_g5_i1 | hyper-polarisation-activated voltage-gated potassium channel |  | upregulated | 1 | regulation of vitamin metabolic process | Y |
| DN71318_c0_g5_i2 | hyper-polarisation-activated voltage-gated potassium channel |  | upregulated | 1 | regulation of vitamin metabolic process | Y |
| DN74481_c13_g2_i1 | putative plastid 1-deoxy-D-xylulose 5-phosphate reducto-isomerase |  | upregulated | 1 | phospholipid biosynthesis, response to cold | N |
| DN70500_c0_g1_i1 | transcription factor YY2 |  | upregulated | 1 | heterocycle biosynthetic process | Y |
| DN74098_c0_g1_i1 | fructose-1,6-bisphosphate aldolase | chloroplast | upregulated | 1 | glycolytic process | Y |
| DN75667_c0_g1_i2 | phospho-glycerate kinase | chloroplast | upregulated | 1 | reductive pentose-phosphate cycle, glycolytic process | N |
| DN74365_c0_g1_i1 | pyridoxal kinase |  | upregulated | 1 | phospholipid biosynthesis | Y |
| DN74365_c0_g1_i2 | pyridoxal kinase |  | upregulated | 1 | phospholipid biosynthesis | Y |
| DN74961_c0_g1_i3 | glycerate translocator | chloroplast | upregulated | 1 | photorespiration, glycolate transmembrance transport, chloroplast assembly | N |
| DN71927_c0_g4_i1 | stem-loop binding protein of 41 kDa b | chloroplast | upregulated | 1 | chloroplast organisation, response to cold | N |
| DN67734_c4_g1_i1 | glycine dehydro-genase | mitochondrion | upregulated | 1 | glycine metabolic process | Y |
| DN71816_c0_g1_i2 | bacterial hit, GNAT fam |  | upregulated | 1 | heterocycle biosynthetic process | Y |
| DN75856_c1_g1_i1 | triose-phosphate/ phosphate translocator | chloroplast | upregulated | 1 | cobalamin metabolism | Y |
| DN73854_c3_g3_i2 | cytochrome b6-f complex iron-sulfur subunit | chloroplast | upregulated | 1 | detection of biotic stimulus, photosystem II assembly, shoot system morphogenesis, regulation of hydrogen peroxide metabolism | N |
| DN72323_c1_g3_i1 | serine hydroxy-methyl-transferase | mitochondrion or cytosolic | upregulated | 1 | photorespiration, glycine metabolic process, response to cold | N |
| DN70627_c0_g2_i2 | putative trans-criptional regulatory protein |  | upregulated | 1 | heterocycle biosynthetic process | Y |
| DN70900_c0_g2_i1 | lysophospho-lipid acyl-transferase |  | upregulated | 1 | phospholipid biosynthesis | Y |
| DN73638_c0_g1_i4 | ABC transporter B family member 5 |  | upregulated | 1 | regulation of protein dephosphorylation | Y |
| DN73611_c0_g3_i1 | PGR5-like protein 1B | chloroplast | upregulated | 1 | heterocycle biosynthetic process | Y |
| DN76651_c3_g2_i3 | green algae hit, glyceralde-hyde-3-phosphate dehydro-genase |  | upregulated | 1 | reductive pentose-phosphate cycle, glycolytic process, negative regulation of telomere maintenance, nucleotide-excision repair, DNA incision,3’-to lesion, cellular response to UV | N |
| DN76706_c0_g2_i2 | cob(I)yrinic acid a,c-diamide adenosyl-transferase |  | upregulated | 1 | heterocycle biosynthetic process, cobalamin metabolism | N |
| DN75126_c0_g1_i3 | coral hit, 7,8 dihydro-8-oxoguanine tri-phosphatase-like |  | upregulated | 1 | heterocycle biosynthetic process | Y |
| DN76392_c0_g1_i2 | probable phospho-lipase D |  | upregulated | 1 | photorespiration | Y |
| DN72560_c0_g2_i1 | magnesium-chelatase subunit ChID | chloroplast | upregulated | 1 | heterocycle biosynthetic process | Y |
| DN77029_c1_g1_i1 | glycine de-carboxylase | mitochondrion | upregulated | 1 | glycine metabolic process | Y |
| DN75024_c0_g4_i1 | PsbP-like protein | chloroplast | upregulated | 1 | heterocycle biosynthetic process, negative regulation of long-day photoperiodism, flowering | N |
| DN74340_c0_g2_i1 | phospho-glycolate phosphatase 1B | chloroplast | upregulated | 1 | photorespiration | Y |
| DN72364_c0_g1_i2 | glycerol-3-phosphate acyl-transferase | chloroplast | upregulated | 1 | phospholipid biosynthesis | Y |
| DN73854_c3_g3_i3 | phospho-ribulokinase | chloroplast | upregulated | 1 | detection of biotic stimulus, regulation of plant-type hypersensitive response, response to cold, response to chitin | N |
| DN76673_c2_g5_i1 | putative transporter |  | upregulated | 1 | heterocycle biosynthetic process, cellular response to UV | N |
| DN74460_c0_g1_i1 | ATP phospho-ribosyl-transferase 2 | chloroplast | upregulated | 1 | heterocycle biosynthetic process | Y |
| DN76376_c1_g1_i3 | cyclin-F |  | upregulated | 1 | heterocycle biosynthetic process, regulation of vitamin metabolic process | N |
| DN74914_c0_g2_i1 | unnamed |  | downregulated | 2 | stress-induced mitochondrial fusion, mitochondrial calcium ion transmembrane transport, positive regulation of mitochondrial membrane potential, positive regulation of mitochondrial DNA replication, mitochondrial protein processing, positive regulation of cardiolipin metabolic process, interleukin-2 production, CD4-positive alpha-beta T cell activation | N |
| DN74914_c0_g2_i2 | stomatin prohibition-family | mitochondrion | downregulated | 2 | stress-induced mitochondrial fusion, mitochondrial calcium ion transmembrane transport, positive regulation of mitochondrial membrane potential, positive regulation of mitochondrial DNA replication, mitochondrial protein processing, positive regulation of cardiolipin metabolic process, interleukin-2 production, CD4-positive alpha-beta T cell activation | N |
| DN74914_c0_g2_i3 | stomatin prohibition-family | mitochondrion | downregulated | 2 | stress-induced mitochondrial fusion, mitochondrial calcium ion transmembrane transport, positive regulation of mitochondrial membrane potential, positive regulation of mitochondrial DNA replication, mitochondrial protein processing, positive regulation of cardiolipin metabolic process, interleukin-2 production, CD4-positive alpha-beta T cell activation | N |
| DN74986_c0_g1_i1 | NAD+ kinase |  | downregulated | 2 | NADP metabolism | Y |
| DN71837_c1_g1_i2 | CCR4-NOT transcription complex |  | downregulated | 2 | nuclear-transcribed mRNA poly(A) tail shortening, RNA phosphodiester bond hydrolysis, exonucleolytic, gene silencing by miRNA | N |
| DN71837_c1_g1_i9 | CCR4-NOT transcription complex |  | downregulated | 2 | nuclear-transcribed mRNA poly(A) tail shortening, RNA phosphodiester bond hydrolysis, exonucleolytic, gene silencing by miRNA | N |
| DN76337_c0_g1_i4 | no hits |  | downregulated | 2 | nuclear-transcribed mRNA poly(A) tail shortening, RNA phosphodiester bond hydrolysis, exonucleolytic, gene silencing by miRNA | N |
| DN74965_c0_g4_i1 | glucose-6-phosphate 1-dehydro-genase | chloroplast | downregulated | 2 | NADP metabolism | Y |
| DN76337_c1_g1_i1 | no hits |  | downregulated | 2 | nuclear-transcribed mRNA poly(A) tail shortening, RNA phosphodiester bond hydrolysis, exonucleolytic, gene silencing by miRNA | N |
| DN69846_c0_g1_i5 | chaperone protein dnaJ |  | downregulated | 2 | chorion development | Y |
| DN69846_c0_g1_i2 | chaperone protein dnaJ |  | downregulated | 2 | chorion development | Y |
| DN76627_c1_g1_i1 | stomatin 2 | mitochondrion | downregulated | 2 | stress-induced mitochondrial fusion, mitochondrial calcium ion transmembrane, positive regulation of mitochondrial DNA replication transport, positive regulation of mitochondrial membrane potential, mitochondrial protein processing, positive regulation of cardiolipin metabolic process | N |
| DN69532_c0_g1_i2 | transaldolase |  | downregulated | 2 | NADP metabolism | Y |

Table S4. Definition for abbreviations found in Figure 4, conceptual model of *Porolithon* cf. *onkodes* cell.

| Abbreviation | Definition |
| --- | --- |
| PGA | Phosphoglycolate phosphatase |
| PGLGG1 | Plastidal glycolate/glycerate translocator |
| PPP | Pentose phosphate pathway |
| G6PDH | Glucose-6-phosphate 1-dehydrogenase |
| 6PGL | 6-phosphogluconolactonase |
| 6PGDH | 6-phosphogluconate dehydrogenase |
| RPI | Ribose 5-phosphate isomerase |
| TK | Transketolase |
| TAL | Transaldolase |
| SH17BPase | Sedoheptulose 1,7-biphosphatase |
| PRK | Phosphoribulokinase |
| RuBisCO | Ribulose-1,5-biphosphate carboxylase/oxygenase |
| PGK | Phosphoglycerate kinase |
| GAPDH | Glyceraldehyde-3 phosphate dehydrogenase |
| TPI | Triosephosphate isomerase |
| FBA | Fructose-bisphosphate aldolase |
| TPT | Triose phosphate/phosphate translocator |
| P_i_ | Inorganic phosphate |
| PGR5 | Proton gradient regulation 5 |

Table S5. Summary of mean carbonate chemistry in the experimental treatments. *p*CO_2_, HCO_3-_, and CO_3_^2-^ were calculated using the R package seacarb by inputting measured pH_T_, total alkalinity (TA), temperature (Temp ºC), and a salinity of 35.5 ± 0.2. All values are mean ± standard error (SE). High-Mg calcite was calculated for 16.4% calcite following methods from G Diaz-Pulido, KRN Anthony, DI Kline, S Dove and O Hoegh-Guldberg [3].

| Treatment [Target] | Temp ºC | pH_T_ | TA µmol kg^-1^ | *p*CO_2_ µatm | HCO_3-_ µmol kg^-1^ | CO_3_^2-^ µmol kg^-1^ | Ω_High-Mg Calcite_ |
| --- | --- | --- | --- | --- | --- | --- | --- |
| 27.2 ºC + pH 8.00 | 27.12 ± 0.060 | 8.00 ± 0.005 | 2291.49 ± 0.653 | 454.19 ± 8.178 | 1777.70 ± 4.330 | 209.14 ± 1.780 | 1.088 ± 0.010 |
| 29.5 ºC + pH 8.00 | 29.29 ± 0.071 | 7.99 ± 0.003 | 2290.71 ± 0.875 | 477.26 ± 3.982 | 1768.71 ± 2.980 | 212.72 ± 1.340 | 1.135 ± 0.030 |
| 27.2 ºC + pH 7.70 | 27.18 ± 0.051 | 7.70 ± 0.002 | 2290.91 ± 0.811 | 1020.44 ± 7.297 | 2005.62 ± 1.860 | 116.54 ± 7.970 | 0.606 ± 0.020 |
| 29.5 ºC + pH 7.70 | 29.48 ± 0.071 | 7.69 ± 0.003 | 2290.31 ± 0.722 | 1028.482 ± 7.076 | 1984.58 ± 1.540 | 125.02 ± 6.850 | 0.672 ± 0.030 |

Table S6. Statistics for number of reads, the number of counts (the counted number of reads mapped to each gene), and mapping % generated during CEL-Seq pipeline for both *Sporolithon* cf. *durum* and *Porolithon* cf. *onkodes*. Samples that had <900,000 reads and low mapping percentage were removed from analysis.

| Species | Sample | # reads | # counts | % mapped |
| --- | --- | --- | --- | --- |
| *S.* cf. *durum* | SD10_amb | 2792473 | 907968 | 67.25% |
| *S.* cf. *durum* | SD3_ph | 2905474 | 999343 | 69.65% |
| *S.* cf. *durum* | SD4_amb | 7230595 | 2078440 | 74.69% |
| *S.* cf. *durum* | SD5_ph | 17392054 | 5225552 | 72.16% |
| *S.* cf. *durum* | SD6_tph | 7571206 | 2565129 | 69.88% |
| *S.* cf. *durum* | SD7_tph | 3042944 | 948133 | 69.44% |
| *S.* cf. *durum* | SD8_temp | 3639126 | 1403593 | 53.51% |
| *S.* cf. *durum* | SD9_ph | 873800 | 262942 | 59.51% |
| *S.* cf. *durum* | SD1_temp | 4455442 | 1745783 | 67.34% |
| *S.* cf. *durum* | SD2_temp | 25755969 | 9499485 | 70.81% |
| *S.* cf. *durum* | SD11_ph | 9239774 | 3644235 | 69.84% |
| *S.* cf. *durum* | SD12_tph | 2052150 | 775382 | 65.55% |
| *S.* cf. *durum* | SD13_amb | 4700800 | 1497574 | 62.54% |
| *S.* cf. *durum* | SD14_ph | 13025010 | 2709167 | 73.76% |
| *S.* cf. *durum* | SD15_tph | 10015974 | 3138109 | 61.05% |
| *S.* cf. *durum* | SD17_amb | 12483294 | 3784547 | 71.55% |
| *S.* cf. *durum* | SD18_temp | 75371988 | 21542260 | 63.91% |
| *S.* cf. *durum* | SD19_tph | 14555346 | 2556820 | 71.85% |
| *S.* cf. *durum* | SD20_temp | 6520222 | 2365847 | 50.66% |
| *P.* cf. *onkodes* | PO10_amb | 11693266 | 2629626 | 49.28% |
| *P.* cf. *onkodes* | PO11_ph | 958480 | 159937 | 47.18% |
| *P.* cf. *onkodes* | PO12_tph | 1813570 | 1256101 | 51.77% |
| *P.* cf. *onkodes* | PO14_ph | 970230 | 177249 | 43.78% |
| *P.* cf. *onkodes* | PO15_tph | 16810993 | 3012292 | 57.63% |
| *P.* cf. *onkodes* | PO16_amb | 2638715 | 532955 | 39.93% |
| *P.* cf. *onkodes* | PO17_amb | 1338205 | 257316 | 51.17% |
| *P.* cf. *onkodes* | PO18_temp | 910868 | 191000 | 50.01% |
| *P.* cf. *onkodes* | PO1_temp | 1338887 | 244611 | 49.36% |
| *P.* cf. *onkodes* | PO2_temp | 13190073 | 2874863 | 50.56% |
| *P.* cf. *onkodes* | PO3_ph | 3767250 | 625636 | 57.24% |
| *P.* cf. *onkodes* | PO4_amb | 1209823 | 205975 | 56.76% |
| *P.* cf. *onkodes* | PO5_ph | 5228592 | 988016 | 49.91% |
| *P.* cf. *onkodes* | PO6_tph | 7691073 | 1530097 | 50.63% |
| *P.* cf. *onkodes* | PO7_tph | 5316967 | 861006 | 60.67% |
| *P.* cf. *onkodes* | PO8_temp | 4164386 | 739996 | 52.81% |

Table S7. Transcripts used for RT-qPCR validation of CEL-Seq expression profiles. Primers for housekeeping (HK) genes and genes of interest (GOI) were designed and used for validation. Table has accession number for transcripts, species, BLASTX annotation of sequence, primer sequences, expected amplicon size (bp), primer optimal annealing temperature (Tm) ºC, and PCR efficiency (%) and coefficient of determination (R^2^) from qPCR standard curve analysis. HK genes were chosen from commonly used HKGs, such as beta-tubulin (β-tubulin), glyceraldehyde 3-phosphate dehydrogenase (GAPDH) and ubiquitin C (UBC), and through investigation of transcripts with low standard deviation and that were not significantly differentially expressed through edgeR analysis (i.e., heme oxygenase).

| Trinity accession number | Species | BLASTX Annotation | Primer sequence (5’-3’)^a^ | Expected amplicon size (bp) | Tm (°C) | PCR efficiency (%) |
| --- | --- | --- | --- | --- | --- | --- |
|  |  |  |  |  |  | (R^2^) |
| DN76782_c6_g1_i1 | *Porolithon* cf. *onkodes* | β-  tubulin (HKG) | (F) TCGGCCCTACTGAGTCGATT | 182 | 57.2 | 99.8 |
|  |  |  | (R) CTGGAGAAGGCATGGACGAG | |  | (0.989) |
| DN95780_c0_g1_i1 | *Porolithon* cf. *onkodes* | heme oxygenase (HKG) | (F) AACCAGAATTACTTGTGTCGCA | 117 | 55.1 | 96 |
|  |  |  | (R) CTGTACCTTCATTGCCAGAAAGT | | | (0.89) |
| DN74877_c1_g1_i1 | *Porolithon* cf. *onkodes* | GAPDH (GOI) | (F) TGTCATTGCTGGCGAGGATT | 175 | 59.7 | 95.5 |
|  |  |  | (R) CTTCGCTCCCGCCTGAATAT | |  | (0.91) |
| DN72227_c0_g4_i1 | *Porolithon* cf. *onkodes* | HSP33 (GOI) | (F) AGGTACGAACTTTGCGGTGT | 166 | 57.2 | 100.1 |
|  |  |  | (R) TGCCAAACCCATGCATTTCG | |  | (0.96) |
| DN73640_c0_g1_i1 | *Porolithon* cf. *onkodes* | photosystem II CP47 (GOI) | (F) CCTATGGACAAAGGCGATGG | 214 | 55.1 | 95 |
|  |  |  | (R) TGACGCTAACACCAACTTGC | |  | (0.99) |
| DN71939_c0_g6_i1 | *Porolithon* cf. *onkodes* | acetyl-CoA (GOI) | (F) ACTCCAACTTCAAACGTGCG | 224 | 59.7 | 120.5 |
|  |  |  | (R) ATCCACATTCACAGCACCGT | |  | (0.87) |
| DN73238_c10_g1_i1 | *Porolithon* cf. *onkodes* | ferritin-3, chloroplastic (GOI) | (F) ATCCACCTTCACAAGCCGAC | 154 | 64 | 85.4 |
|  |  |  | (R) AACGAAAACGAGCCCTGACA | |  | (0.99) |
| DN199936_c0_g1_i2 | *Sporolithon* cf. *durum* | GAPDH (HKG) | (F) ACAACTCGCAGGAAAGCTCA | 212 | 55.1 | 120 |
|  |  |  | (R) TGCTTTCATCGGTGCCCTTA | |  | (0.99) |
| DN198856_c0_g2_i4 | *Sporolithon* cf. *durum* | serine/threonine protein phosphatase (GOI) | (F) ATGCAGGCCCTTGAGTTTGT | 241 | 64 | 109 |
|  |  |  | (R) TGATACCGTTGCTCTGCCAG | |  | (-0.90) |
| DN199578_c0_g1_i2 | *Sporolithon* cf. *durum* | acetyl-CoA (GOI) | (F) AGACGGTGCAGTTGGAGATC | 219 | 59.7 | 109 |
|  |  |  | (R) ATATCCCCACCTTCCGATGC | |  | (0.96) |
| DN196675_c0_g3_i7 | *Sporolithon* cf. *durum* | UBC (HKG) | (F) TGCACAAACTACCTCACGCA | 192 | 57.2 | 88.9 |
|  |  |  | (R) ATGGTGCTCACTTGCTCACA | |  | (-0.94) |
| DN197763_c0_g2_i5 | *Sporolithon* cf. *durum* | HSP33 (GOI) | (F) CATCGGTCCAGGTCACTACG | 223 | 57.2 | 112.4 |
|  |  |  | (R) ATCGCGGCATAGAACTGAGG | |  | (-0.94) |
| ^a^ F, forward primer; R, reverse primer. | | | |  |  |  |

**Additional file 2**

Dataset S1 (separate file). Table of all significantly (FDR < 0.05), differentially expressed genes (DEGs) from pairwise comparisons of experimental treatments from edgeR analysis for *Porolithon cf. onkodes*. Table includes *P. cf. onkodes* gene identifiers and values for log expression fold changes (logFC), log counts per million (logCPM), *F* statistic, *p* value, false discovery rate (FDR; *p* adjusted by the Benjamini-Hochberg procedure) for pairwise comparisons of each treatment combination. Treatment comparisons are listed as follows: control (27.2 ºC + 8.0 pH), T (29.5 ºC + 8.0 pH), pH (27.2 ºC + 7.7 pH), and T+pH (29.5 ºC + 7.7 pH). Annotations for DEGs that returned BLASTX similarity search hits are given. If transcript didn't return a hit, no annotation is given, and cell is left blank.

**References**

1. Dickson AG, Sabine CL, Christian JR: Guide to best practices for ocean CO_2_ measurements: North Pacific Marine Science Organization; 2007.

2. Gattuso J-P, Epitalon J-M, Lavigne H, Orr JC, Gentili B, Hagens M, Hofmann A, Mueller J-D, Proye A, Rae J *et al*: seacarb: seawater carbonate chemistry with R. In*.*, 3.2.12 edn; 2019.

3. Diaz-Pulido G, Anthony KRN, Kline DI, Dove S, Hoegh-Guldberg O: Interactions between ocean acidification and warming on the mortality and dissolution of coralline algae. *Journal of Phycology* 2012, 1(48):32-39.

4. Page TM, McDougall C, Diaz-Pulido G: *De novo* transcriptome assembly for four species of crustose coralline algae and analysis of unique orthologous genes. *Scientific Reports* 2019, 9(1):12611.

5. Hashimshony T, Senderovich N, Avital G, Klochendler A, de Leeuw Y, Anavy L, Gennert D, Li S, Livak KJ, Rozenblatt-Rosen O *et al*: CEL-Seq2: Sensitive highly-multiplexed single-cell RNA-Seq. *Genome Biology* 2016, 17(1):77.

6. McDougall C, Aguilera F, Shokoohmand A, Moase P, Degnan BM: Pearl sac gene expression profiles associated with pearl attributes in the silver-lip pearl oyster, *Pinctada maxima*. *Frontiers in Genetics* 2021, 11:597459-597459.

7. Schmieder R, Edwards R: Quality control and preprocessing of metagenomic datasets. *Bioinformatics* 2011, 27(6):863-864.

8. Langmead B, Trapnell C, Pop M, Salzberg SL: Ultrafast and memory-efficient alignment of short DNA sequences to the human genome. *Genome Biology* 2009, 10(3):R25.

9. Grün D, Kester L, van Oudenaarden A: Validation of noise models for single-cell transcriptomics. *Nature Methods* 2014, 11(6):637-640.

10. Robinson MD, McCarthy DJ, Smyth GK: edgeR: a Bioconductor package for differential expression analysis of digital gene expression data. *Bioinformatics* 2010, 26(1):139-140.

11. Kolde R: pheatmap: Pretty heatmaps. In*.* Edited by Kolde R, 1.0.12 edn; 2015.

12. Shannon P, Markiel A, Ozier O, Baliga NS, Wang JT, Ramage D, Amin N, Schwikowski B, Ideker T: Cytoscape: A software environment for integrated models of biomolecular interaction networks. *Genome Research* 2003, 13(11):2498-2504.

13. Heymans K, Kuiper M, Maere S: BiNGO: a Cytoscape plugin to assess overrepresentation of Gene Ontology categories in Biological Networks. *Bioinformatics* 2005, 21(16):3448-3449.

14. Supek F, Bošnjak M, Škunca N, Šmuc T: REVIGO summarizes and visualizes long lists of gene ontology terms. *PLOS ONE* 2011, 6(7):e21800.

15. Altschul SF, Gish W, Miller W, Myers EW, Lipman DJ: Basic local alignment search tool. *Journal of Molecular Biology* 1990, 215:403-410.

16. Kanehisa M, Sato Y, Kawashima M, Furumichi M, Tanabe M: KEGG as a reference resource for gene and protein annotation. *Nucleic Acids Research* 2016, 44(D1):D457-D462.

17. Li B, Chen C, Xu Y, Ji D, Xie C: Validation of housekeeping genes as internal controls for studying the gene expression in *Pyropia haitanensis* (Bangiales, Rhodophyta) by quantitative real-time PCR. *Acta Oceanologica Sinica* 2014, 33(9):152-159.

18. Zeng J, Liu S, Zhao Y, Tan X, Aljohi HA, Liu W, Hu S: Identification and analysis of house-keeping and tissue-specific genes based on RNA-seq data sets across 15 mouse tissues. *Gene* 2016, 576(1, Part 3):560-570.

19. Rozen S, Skaletsky H: Primer3 on the WWW for general users and for biologist programmers. *Methods Mol Biol* 2000, 132:365-386.

20. Vandesompele J, De Preter K, Pattyn F, Poppe B, Van Roy N, De Paepe A, Speleman F: Accurate normalization of real-time quantitative RT-PCR data by geometric averaging of multiple internal control genes. *Genome Biology* 2002, 3(7):1-12.

21. Page TM, Diaz-Pulido G: Plasticity of adult coralline algae to prolonged increased temperature and *p*CO_2_ exposure but reduced survival in their first generation. *PLOS ONE* 2020, 15(6):e0235125.

22. Marsh Jr JA: Primary productivity of reef‐building calcareous red algae. *Ecology* 1970, 51(2):255-263.

23. Peña V, Vieira C, Carlos Braga J, Aguirre J, Rösler A, Baele G, De Clerck O, Le Gall L: Radiation of the coralline red algae (Corallinophycidae, Rhodophyta) crown group as inferred from a multilocus time-calibrated phylogeny. *Molecular Phylogenetics and Evolution* 2020, 150:106845.

24. Anthony KRN, Kline DI, Diaz-Pulido G, Dove S, Hoegh-Guldberg O: Ocean acidification causes bleaching and productivity loss in coral reef builders. *Proceedings of the National Academy of Sciences* 2008, 105(45):17442-17446.

25. Bergstrom E, Ordoñez A, Ho M, Hurd C, Fry B, Diaz-Pulido G: Inorganic carbon uptake strategies in coralline algae: Plasticity across evolutionary lineages under ocean acidification and warming. *Marine Environmental Research* 2020, 161:105107.

26. Kim J-H, Kim N, Moon H, Lee S, Jeong SY, Diaz-Pulido G, Edwards MS, Kang J-H, Kang EJ, Oh H-J *et al*: Global warming offsets the ecophysiological stress of ocean acidification on temperate crustose coralline algae. *Marine Pollution Bulletin* 2020, 157:111324.

27. Martin S, Cohu S, Vignot C, Zimmerman G, Gattuso J-P: One-year experiment on the physiological response of the Mediterranean crustose coralline alga, *Lithophyllum cabiochae*, to elevated *p*CO_2_ and temperature. *Ecology and Evolution* 2013, 3(3):676-693.

28. Qui-Minet ZN, Coudret J, Davoult D, Grall J, Mendez‐Sandin M, Cariou T, Martin S: Combined effects of global climate change and nutrient enrichment on the physiology of three temperate maerl species. *Ecology and Evolution* 2019, 9(24):13787-13807.

29. Vásquez-Elizondo RM, Enríquez S: Coralline algal physiology is more adversely affected by elevated temperature than reduced pH. *Scientific Reports* 2016, 6:19030.

30. Sordo L, Santos R, Barrote I, Silva J: Temperature amplifies the effect of high CO_2_ on the photosynthesis, respiration, and calcification of the coralline algae *Phymatolithon lusitanicum*. *Ecology and Evolution* 2019, 9(19):11000-11009.

31. Muñoz PT, Sáez CA, Martínez-Callejas MB, Flores-Molina MR, Bastos E, Fonseca A, Gurgel CFD, Barufi JB, Rörig L, Hall-Spencer JM *et al*: Short-term interactive effects of increased temperatures and acidification on the calcifying macroalgae *Lithothamnion crispatum* and *Sonderophycus capensis*. *Aquatic Botany* 2018, 148:46-52.
